# (KAUH-BCMD) dataset: advancing mammographic breast cancer classification with multi-fusion preprocessing and residual depth-wise network

**DOI:** 10.3389/fdata.2025.1529848

**Published:** 2025-03-06

**Authors:** Asma'a Mohammad Al-Mnayyis, Hasan Gharaibeh, Mohammad Amin, Duha Anakreh, Hanan Fawaz Akhdar, Eman Hussein Alshdaifat, Khalid M. O. Nahar, Ahmad Nasayreh, Mohammad Gharaibeh, Neda'a Alsalman, Alaa Alomar, Maha Gharaibeh, Hamad Yahia Abu Mhanna

**Affiliations:** ^1^Department of Internal Medicine, College of Medicine, Yarmouk University, Irbid, Jordan; ^2^Artificial Intelligence and Data Innovation Office, King Hussein Cancer Center, Amman, Jordan; ^3^Computer Science Department, Faculty of Information Technology and Computer Sciences, Yarmouk University, Irbid, Jordan; ^4^Department of Medicine, Faculty of Medicine, Jordan University of Science and Technology, Irbid, Jordan; ^5^Physics Department, Imam Mohammad Ibn Saud Islamic University (IMSIU), Riyadh, Saudi Arabia; ^6^Department of Obstetrics and Gynecology, Faculty of Medicine, Yarmouk University, Irbid, Jordan; ^7^Department of Medicine, Faculty of Medicine, Hashemite University, Zarqa, Jordan; ^8^Department of Computer Science, Faculty of Information Technology, Jordan University of Science and Technology, Irbid, Jordan; ^9^Radiology Department, Jordan University of Science and Technology, Irbid, Jordan; ^10^School of Physics, Universiti Sains Malaysia (USM), Penang, Malaysia

**Keywords:** mammogram dataset, residual network, CNN, deep learning, processing images, breast cancer

## Abstract

The categorization of benign and malignant patterns in digital mammography is a critical step in the diagnosis of breast cancer, facilitating early detection and potentially saving many lives. Diverse breast tissue architectures often obscure and conceal breast issues. Classifying worrying regions (benign and malignant patterns) in digital mammograms is a significant challenge for radiologists. Even for specialists, the first visual indicators are nuanced and irregular, complicating identification. Therefore, radiologists want an advanced classifier to assist in identifying breast cancer and categorizing regions of concern. This study presents an enhanced technique for the classification of breast cancer using mammography images. The collection comprises real-world data from King Abdullah University Hospital (KAUH) at Jordan University of Science and Technology, consisting of 7,205 photographs from 5,000 patients aged 18–75. After being classified as benign or malignant, the pictures underwent preprocessing by rescaling, normalization, and augmentation. Multi-fusion approaches, such as high-boost filtering and contrast-limited adaptive histogram equalization (CLAHE), were used to improve picture quality. We created a unique Residual Depth-wise Network (RDN) to enhance the precision of breast cancer detection. The suggested RDN model was compared with many prominent models, including MobileNetV2, VGG16, VGG19, ResNet50, InceptionV3, Xception, and DenseNet121. The RDN model exhibited superior performance, achieving an accuracy of 97.82%, precision of 96.55%, recall of 99.19%, specificity of 96.45%, F1 score of 97.85%, and validation accuracy of 96.20%. The findings indicate that the proposed RDN model is an excellent instrument for early diagnosis using mammography images and significantly improves breast cancer detection when integrated with multi-fusion and efficient preprocessing approaches.

## 1 Introduction

Breast cancer is the most frequent malignancy diagnosed in women, ranking among the three most common cancers worldwide (Harbeck and Gnant, 2017). With an estimated 2.3 million new cases, accounting for 11.6% of all cancer cases, and 666,000 deaths, which was ~6.9% of all cancer deaths that involved women in 2022 (Bray et al., 2024). This disease occurs when the breast tissue grows abnormally and divides uncontrollably, resulting in excessive growth of cells and leading to the formation of a tumor (Balakumaran et al., 2010). The etiology of breast cancer is attributed to a combination of multiple risk factors, these include a previous diagnosis of breast cancer, genetic factors as positive family history, some hormonal risk factors including, nulliparity, early menarche, delayed menopause, the use of hormone replacement therapy, as well as environmental factors as tobacco use, alcohol intake, obesity and sedentary lifestyle, Conversely, factors linked to a reduced risk of developing breast cancer are multiparity, a history of breastfeeding, regular physical activity, and weight loss (Admoun and Mayrovitz, 2022).

The clinical manifestation of breast cancer can vary widely, ranging from asymptomatic breast masses to palpable lumps, skin changes, or nipple discharge (Sung et al., 2021). An early detection of breast cancer can significantly improve the disease treatment outcomes and increase the survival rate by 85% (Balakumaran et al., 2010; Zeeshan et al., 2018). The diagnostic approach of a breast lesion is performed using a triple assessment, a standardized method consisting of three critical components: clinical breast examination, imaging studies, and biopsy (Nigam and Nigam, 2013). The utilization of imaging modalities is a fundamental approach for the detection, assessment, and response to therapy in patients with breast cancer (Jafari et al., 2018). Among the most used methods in the decade is mammography. A technique that requires a radiologist's careful examination to detect potential breast cancer characteristics (Kalpana and Selvy, 2024).

A mammogram is an X-ray machine that emits a short burst of X-rays that passes through the breast tissue to a detector on the opposite side. The detector can be either a photographic film plate, which records the x-ray image on film, or a solid-state detector, that sends electronic impulses to a computer, resulting in a digital image (Screening and Board, 2024). Other imaging modalities include ultrasonography (US) and magnetic resonance imaging (MRI) while these techniques have their benefits, mammograms remain the gold standard for breast cancer screening for multiple reasons. Firstly, Mammograms are particularly sensitive in detecting subtle abnormalities in breast tissue that may be missed by other imaging techniques. Secondly, mammograms offer an excellent spatial resolution, allowing for precise localization of detected lesions. These factors contribute to the continued dependence on mammograms as the primary screening tool for breast cancer (Screening and Board, 2024).

On the other hand, literature has shown several limitations facing human radiologists in reading mammograms, with reported sensitivity and specificity rates ranging from 77% to 87% and 89% to 97%, respectively (Ribli et al., 2018). As a consequence, Double reading has been advocated in the majority of screening programs, leading to a significant increase in the time burden on human radiologists (Suh et al., 2020). Furthermore, a notable constraint referred to as tissue overlap occurs when distinct breast tissues, merely separated by projection direction, are represented at the same position in the 2D mammographic image. Consequently, normal tissues may conceal the presence of a malignant disease, diminishing sensitivity. The representation of unique normal tissues may resemble a concerning lesion, hence diminishing specificity. Their effects considerably reduce the precision of 2D mammography, particularly in breasts with substantial fibro-glandular tissue (i.e., thick breasts), which occurs in roughly fifty percent of the screened population and accounts for one-third of undetected cancers (Sechopoulos et al., 2021).

These challenges and limitations have spurred the exploration of deep-learning techniques for medical image analysis (Kalpana and Selvy, 2024). The recent improvements in digital imaging, imaging modalities, and computer development have led to increased interest in breast cancer research area (Mokni et al., 2021). Using AI-based computer-aided detection (CAD) systems allows for the analysis of medical images using machine learning techniques and advanced algorithms to detect potential anomalies. These systems can process large volumes of data efficiently, detect subtle patterns that may be missed by the human radiologist, and provide objective assessments (Liu et al., 2023). The proposed Machine learning-based CAD system can help radiologists interpret these images more accurately, thereby enhancing breast cancer's diagnostic process and supporting faster patient care decision-making (Alshammari et al., 2021). Results from a study by demonstrated that an AI-CAD system could accurately differentiate between benign and malignant breast lesions, even in challenging cases (Nasser and Yusof, 2023). In addition, a meta-analysis by found that AI-CAD systems significantly improved the detection of breast cancer by achieving a sensitivity of 93.1%, and specificity of 68.7% when compared to human interpretation alone (Liu et al., 2023).

The study's contributions include the following:

- A breast cancer mammography dataset was collected from King Abdullah University Hospital and is referred to in this research as (KAUH-BCMD).
- The collected data was compared to breast cancer mammography image datasets and is described in Section 2.4.
- Several fusion methods, including high-enhanced filtering and contrast-limited adaptive histogram equalization (CLAHE), have been employed to improve image quality.
- To improve breast cancer diagnosis accuracy, a novel residual depth network (RDN) is created that blends residual learning with depth-separable convolutions to improve computational efficiency and accuracy. This design employs an inverted residual bottleneck architecture, which significantly extends the feature space while requiring a minimal number of parameters.
- The model used in this research was compared with several pre-trained models.

The rest of this work is organized as follows. Section 2 covers related classification research. Section 3 discusses the basic strategies and methods used in the proposed scheme. Experimental results are presented in Section 4, along with a comparison of the proposed strategy with existing methods. Section 5 summarizes the conclusions we have reached through our investigation and Section 6 provides a plan for our future work.

## 2 Literature review

Breast cancer diagnosis has been gradually improved by the application of deep learning models across various forms of medical imaging, including mammography, ultrasound, and histology. This section examines related research on CNN architecture, hybrid models, and pre-trained models that have achieved high classification accuracy in breast cancer detection, demonstrating the effectiveness of integrating diverse data and improving feature extraction procedures.

### 2.1 Deep learning and machine learning in breast cancer diagnosis

Breast cancer diagnosis, especially in resource-limited settings, despite challenges like false positives. Recent AI applications focus on lesion detection, segmentation, classification, and cancer risk prediction, addressing limitations and exploring prospects in medical imaging (Vocaturo and Zumpano, 2021). Deep learning has revolutionized breast cancer image analysis by enabling segmentation, feature extraction, classification, and detection directly from raw medical images across various modalities, including ultrasound, mammography, MRI, and digital breast tomosynthesis (Debelee et al., 2020). Machine Learning and Deep Learning techniques in breast cancer detection and classification using various imaging modalities like mammography, histopathology, ultrasound, and MRI. potential, limitations, and future directions of AI in clinical decision-making for breast cancer diagnosis, emphasizing the need for external validations and accessible datasets (Dar et al., 2022).

In Gagliardi et al. (2024a) convolutional neural networks (CNNs), including variants like ResNet, VGG, and Inception models, for breast ultrasound image classification. These models achieved accuracies between 85% and 88%, often requiring substantial training times or relying on small datasets, which limited generalization. While Gagliardi et al. (2024b) focuses on leveraging deep learning models for breast ultrasound image analysis, highlighting their dual capability in classification and segmentation. Using the BUSI dataset, the study evaluates multiple models, identifying the best-performing one with over 90% accuracy, 92% precision, 90% recall, and a 90% F1 score. The proposed method enhances diagnostic effectiveness by providing tumor mass masks alongside classifications, demonstrating potential for clinical application.

In Shahid and Imran (2025) reviews the advancements in deep learning for breast cancer detection, highlighting its potential to achieve accuracies up to 93.8%, surpassing traditional methods. It also discusses challenges, emerging trends, and future directions for improving breast cancer diagnosis using AI. In Mahesh et al. (2025) introduces an optimized framework using EfficientNetB7 and targeted augmentation strategies to improve breast ultrasound image classification, achieving 98.29% accuracy. The approach addresses overfitting, image distortions, and class imbalances, offering a robust tool for early breast cancer detection. While Manna et al. (2025) proposes the GradeDiff-IM model, combining multiple ML models and deep learning for cancer grade classification, achieving accuracies of 98.2% for G1, 97.6% for G2, and 97.5% for G3. The stacking ensemble approach outperforms single ML and DL models, improving grade classification accuracy.

The Keogan (2025) uses Fourier-transform infrared (FTIR) chemical images of breast cancer tissue to train deep learning models for predicting disease recurrence, achieving an ROC AUC of 0.64. The results suggest that all-digital chemical imaging offers a promising, label-free approach for histopathological prognosis in breast cancer. In Natarajan et al. (2025) proposes the Dynamic Harris Hawks Optimized Gated Recurrent Unit (DHH-GRU) framework for breast cancer prediction, achieving 98.05% accuracy on the Wisconsin Diagnostic Breast Cancer dataset. By combining GRU for temporal data and Harris Hawks Optimization, the method demonstrates superior performance compared to existing techniques.

### 2.2 CNN on mammogram beast dataset

These studies illustrate many advanced techniques for identifying breast cancer in mammograms through deep learning methodologies. The initial work employs a CNN model and preprocessing techniques to develop a deep learning system that precisely and effectively classifies lesions in mammograms as malignant or non-malignant. The MIAS dataset had sensitivity, specificity, accuracy, and AUC of 98%, 92.6%, 95.3%, and 0.974%, respectively, whereas the INbreast dataset exhibited values of 96.55%, 96.49%, 96.52%, and 0.98%, respectively (El Houby and Yassin, 2021). The second study introduces a deep bottleneck convolutional neural network optimized with Bayesian techniques, yielding a maximum accuracy of 96.5% with a sensitivity rate of 96.45% when tested on the INbreast dataset, demonstrating high precision in breast cancer diagnosis (Jabeen et al., 2024). In another approach, a CNN optimized with Particle Swarm Optimization (PSO) achieved success rates of 98.23% and 97.98% on the DDSM and MIAS datasets, respectively, highlighting the effectiveness of this technique for automated predictions (Aguerchi et al., 2024). Additionally, a computer-aided diagnosis model that combines CNNs with a pruned ensembled extreme learning machine (HCPELM) achieved an accuracy of 86% using the MIAS dataset, surpassing other benchmark models and showcasing its utility in early detection (Sureshkumar et al., 2024). Finally, the Fusion of Hybrid Deep Features (FHDF) approach, which combines multiple CNN architectures like VGG16, VGG19, ResNet50, and DenseNet121, attained maximum accuracy rates of 98.706%, 97.734%, and 98.834% on the MIAS, CBIS-DDSM, and INbreast datasets, respectively, proving to be a robust solution for early tumor detection (Al-Hejri, 2024). Collectively, these studies highlight the significant advancements in deep learning techniques for enhancing breast cancer classification and diagnosis.

### 2.3 Hybrid models on mammogram breast dataset

This compilation of research illustrates various innovative methodologies in breast cancer detection utilizing machine learning and deep learning techniques. The first study introduces Quantum SpinalNet (Q-SpinalNet), which employs a Non-Local Means Filter for preprocessing and ETSegNet for segmentation of mammogram images, achieving an accuracy of 90.3%, with a True Negative Rate (TNR) of 90.9% and a True Positive Rate (TPR) of 90% (Sathish, 2024). Another study emphasizes the application of artificial intelligence in digital mammography, utilizing Haar wavelet feature extraction followed by classification with a hybrid deep neural network, achieving an area under the curve (AUC) of 0.92 for early breast cancer detection (Karthiga et al., 2024). In a different approach, a hybrid model combining the quantum-inspired binary Grey Wolf Optimizer (IQI-BGWO) with Support Vector Machine (SVM) techniques, tested on the MIAS dataset, achieved remarkable performance metrics: a mean accuracy of 99.25%, sensitivity of 98.96%, and specificity of 100% (Bilal et al., 2024). Addressing the pressing need for effective diagnostic systems, another study proposes an automated computer-aided diagnosis system that enhances mammogram contrast and employs the EfficientNet-B4 architecture, yielding classification accuracies of 98.459% on the INbreast database and 96.175% on the CBIS-DDSM database (Chakravarthy et al., 2024). Finally, a novel neural network approach combines feature extraction from AlexNet and ResNet18 to improve the classification and segmentation of mammographic images, demonstrating enhanced accuracy in differentiating between benign and malignant cases, trained on a dataset of 2,138 images (Makovetskii et al., 2024). Together, these studies highlight the rapid advancements in computational techniques aimed at enhancing breast cancer diagnosis and improving patient outcomes.

### 2.4 Pre-trained model on mammogram breast dataset

This collection of research highlights significant advancements in breast cancer detection methodologies utilizing various machine learning and deep learning techniques. The first study introduces a breast cancer diagnosis model based on 3D mammography images, achieving a remarkable accuracy of 96.6% through preprocessing and segmentation methods, notably employing the Adaptive Thresholding with Region Growing Fusion Model (AT-RGFM) optimized by the Modified Garter Snake Optimization Algorithm (MGSOA) (Umamaheswari and Babu, 2024). In another approach, a Convolutional Neural Network (CNN) model based on the VGG16 architecture showcases an impressive average identification rate of 96.945% for classifying breast X-ray mammography images into benign and malignant categories, utilizing data collected from the Medical Imaging Department of Ganzhou People's Hospital and Jinan University's Sixth Affiliated Hospital (Liu et al., 2024). Further, a novel detection method leveraging the ResNet50 framework combined with heat mapping and Grad-Cam visualization achieved an accuracy of 0.8920 on the FDDM dataset and an even higher 0.9830 on the MIAS dataset (Gharaibeh et al., 1943). Another study emphasizes a dual-branch model that processes mammograms from both Cranial Caudal (CC) and Mediolateral Oblique (MLO) views, attaining a top accuracy of 95.86% using the CBIS-DDSM dataset, indicating significant improvements in breast cancer classification capabilities (Boudouh and Bouakkaz, 2024). Lastly, the introduction of StethoNet, a deep learning framework trained on the Chinese Mammography Database (CMMD), demonstrated robust performance across multiple datasets, achieving AUC values of 90.7% for CMMD, 83.9% for INbreast, and 85.7% for Vindr-Mammo (Lamprou et al., 2024). Collectively, these studies underscore the potential of integrating advanced computational techniques to enhance early detection and diagnosis of breast cancer, ultimately contributing to improved patient outcomes.

### 2.5 A some of the current mammography repositories

Both public and private mammography databases are used by breast cancer researchers. In this section, a few frequently used mammography datasets are briefly reviewed.

#### 2.5.1 The dataset of the mammographic image analysis society (MIAS)

Among the oldest is the MIAS dataset. The dataset is proprietary and owned by a UK research organization. There are 161 instances and 322 photos encompassing benign, malignant, and normal mammograms. Images with annotations depicting circles around the region of interest are provided in the file (Suckling et al., 2015).

#### 2.5.2 The INbreast dataset

The breast research group rendered its mammography dataset, termed INBREAST, freely available. The data, produced by the Breast Center at CHSJ Porto Hospital of St. John (CHSJ), was released in 2010. The study comprised a total of 410 photos, consisting of 115 DICOM-formatted examples and 90 photographs captured from two perspectives (CC, MLO). Twenty-five examples (two photographs for each case) were sourced from patients who underwent breast surgery, whereas 90 cases (four images per case) were derived from women who had bilateral breast procedures. The BI-RADS algorithm was used to classify bulk, calcification, and normal pictures in the INBREAST dataset. The dataset (Moreira et al., 2012) is no longer accessible.

#### 2.5.3 The curated breast imaging subset (CBIS-DDSM) dataset

The CBIS-DDSM dataset represents an enhanced iteration of the DDSM (Heath et al., 1998). The objective of this dataset is to improve DDSM picture segmentation. The CBIS-DDSM evaluates segmentation methodologies and revises ROI annotations. Breast cancer detection models may be trained and evaluated using a dataset of over 1,000 images, categorized into two types of abnormalities: mass and calcification (Lee et al., 2017).

#### 2.5.4 The CSAW-S dataset

The CSAW-S dataset (Matsoukas et al., 2020) addresses the challenge of limited data by providing 342 mammograms annotated with expert radiologist labels for cancer, along with additional non-expert annotations of breast anatomy (e.g., skin, pectoral muscle, nipple). While these non-expert labels may be imperfect, they significantly enhance segmentation performance when combined with expert annotations. Experiments demonstrate that a network trained solely on expert labels is outperformed by one that incorporates these complementary non-expert annotations, thus transforming the task into a multi-class problem.

#### 2.5.5 The King Abdulaziz University Breast Cancer Mammogram Dataset (KAU-BCMD)

The first extensive dataset in Saudi Arabia that focuses on a significant number of mammography scans is the King Abdulaziz University Breast Cancer Mammography Dataset (KAU-BCMD) (Alsolami et al., 2021). Gathered from King Abdulaziz University's Sheikh Mohammed Hussein Al-Amoudi Center of Excellence in Breast Cancer, it contains 1,416 cases, each of which has two views of the left and right breasts, for a total of 5,662 mammogram images according to the Breast Imaging Reporting and Data System (BIRADS). Three seasoned radiologists have carefully commented and examined it, making it a useful resource that covers a range of imaging modalities and cancer grades pertinent to Saudi women.

#### 2.5.6 The Digital Mammography Dataset For Breast Cancer Diagnosis Research (DMID)

The Digital Mammography Dataset for Breast Cancer Diagnosis Research (DMID) (Oza et al., 2024) contains 225 cases with a total of 510 mammographic images. Each image is annotated for breast mass segmentation analysis, making this dataset a valuable resource for researchers focusing on breast cancer diagnosis and detection. It contains many images. Table 1 provides a comparison between different mammography datasets.

**Table 1:** A summary of the mammography datasets.

**Dataset**	**MIAS (Suckling et al., 2015)**	**INbreast (Moreira et al., 2012)**	**(CBIS-DDSM) (Lee et al., 2017)**	**CSAW-S (Matsoukas et al., 2020)**	**(KAU-BCMD) (Alsolami et al., 2021)**	**(DMID) (Oza et al., 2024)**	**(KAUH-BCMD)**
Original	UK	Portugal	USA	Sweden	KSA	India	Jordan
Year	1994	2010	2017–2018	2020	2021	2023	2024
Number of cases	161 cases	115 cases	6,775 cases	172 cases	1,416 cases	225 cases	5,000 cases
Number of images	322 images	410 images	10,239 images	338 images	5,662 images	510 images	7,205 images
Views	MLO	MLO, CC	MLO, CC	NA	MLO, CC	MLO, CC	MLO, CC
Image type file	PGM	DICOM, XML	DICOM	DICOM	DICOM and JPG formats	DICOM, TIFF	DICOM and JPG formats
BI-RADS	NO	YES	YES	YES	YES	YES	NO
Ground truth	YES	NO	YES	YES	YES	YES	YES
Patient information	NO	YES	YES, Age	NO	YES, Age	NO	YES, Age
Dataset type	Private	Public	Public	Public	Public	Public	Public

#### 2.5.7 Other datasets

The MIRacle dataset (Antoniou et al., 2009) comprises mammography pictures from radiologists, which are employed for machine learning purposes. Two hundred-four photos from 169 cases are presented. This dataset has two modalities: radiologist evaluation and classification. The Italian Magic 5 dataset was compiled from multiple hospitals. There are 967 cases, contingent upon the pathogenic kind (Tangaro et al., 2008). A digital mammography dataset from the radiology department of the university hospital in Nijmegen, Netherlands, was released, although it is currently inaccessible (Karssemeijer et al., 2012). A total of 197 images in two perspectives that were saved in image cytometry standard (ICS) format make up the LLNL dataset (Karssemeijer et al., 2012). The file also contains biopsy results and patient information. The IRAM dataset (Oliveira et al., 2008) integrates multiple datasets. It has numerous images. Table 2 presents a comparison of various mammography datasets. The academic community has access to around 25% of mammography datasets.

**Table 2:** A summary of the mammography datasets.

**Dataset**	**Nijmegen (Karssemeijer et al., 2012)**	**Magic5 (Tangaro et al., 2008)**	**Trueta (Oliver et al., 2010)**	**IRAM (Oliveira et al., 2008)**	**Malaga (Oliver et al., 2010)**	**LLNL (Karssemeijer et al., 2012)**	**MIRacle (Antoniou et al., 2009)**	**(KAUH-BCMD)**
Original	Netherlands	Italian	Spain	Germany	Spain	USA	Greece	Jordan
Year	1998	2002	2008	2008	2008	2008	2009	2024
Number of cases	21 cases	967 cases	89 cases	NA cases	35 cases	50 cases	196 cases	5,000 cases
Number of images	40 images	3,369 images	320 images	10,500 images	NA images	198 images	204 images	7,205 images
Views	MLO, CC	MLO, CC	MLO, CC	MLO, CC	MLO, CC	MLO, CC	NA	MLO, CC
Image type file	NA	DICOM	DICOM	Several	Raw	ICS	NA	DICOM and JPG formats
BI-RADS	NO	NO	YES	YES	NA	NA	YES	NO
Ground truth	YES	YES	YES	NO	NO	NO	YES	YES
Patient information	NA	YES, Age	NA	NA	NA	NO	NO	YES, Age
Dataset type	Private	Private	Private	Private	Private	Private	Private	Public

## 3 Methodology

**Figure 1:**
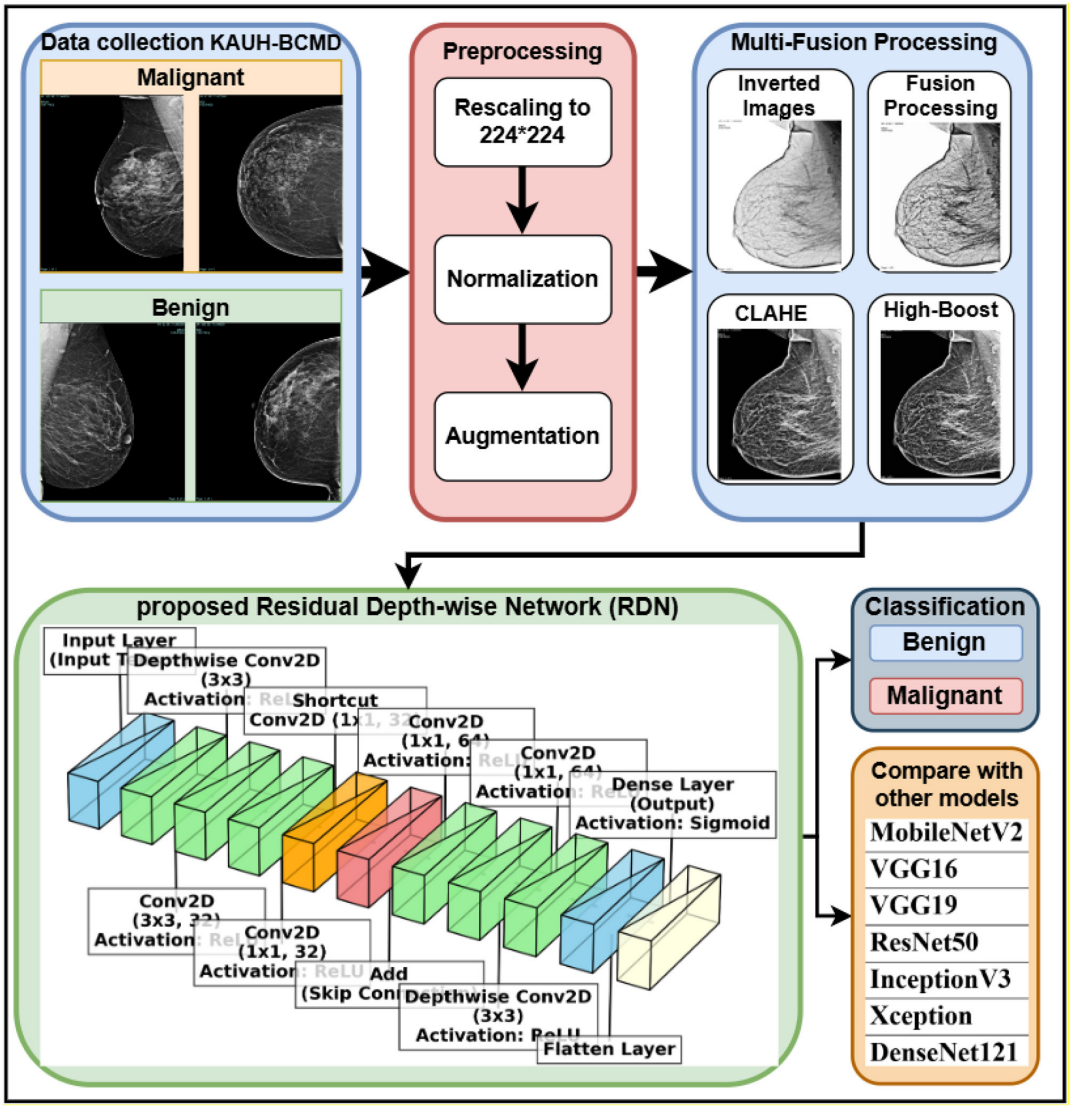
Methodology workflow.

This section outlines a thorough approach of classifying breast cancer using information obtained from King Abdullah University Hospital. The input photographs underwent preprocessing to enhance quality and consistency, including many phases including rescaling, normalization, and augmentation. A multi-fusion image processing technique was used to enhance feature extraction, including inversion, adaptive CLAHE histogram equalization, and high-boost filtering. The classification model used a proposed Residual Depth-wise Network (RDN), integrating residual learning with depth-wise separable convolutions to enhance computational efficiency and precision. This design has an inverted residual bottleneck architecture, which considerably enlarges the feature space while using a few parameters. The integration of these advanced methodologies enhances model efficacy while reducing resource requirements, making it a viable choice for breast cancer diagnosis in medical imaging. Figure 1 illustrates the research process and the approaches used for the training and testing of breast cancer diagnosis (benign and malignant). The ultimate prediction differentiates between benign and malignant breast tissue.

### 3.1 KAUH-BCMD dataset

#### 3.1.1 Mammography data description

**Figure 2:**
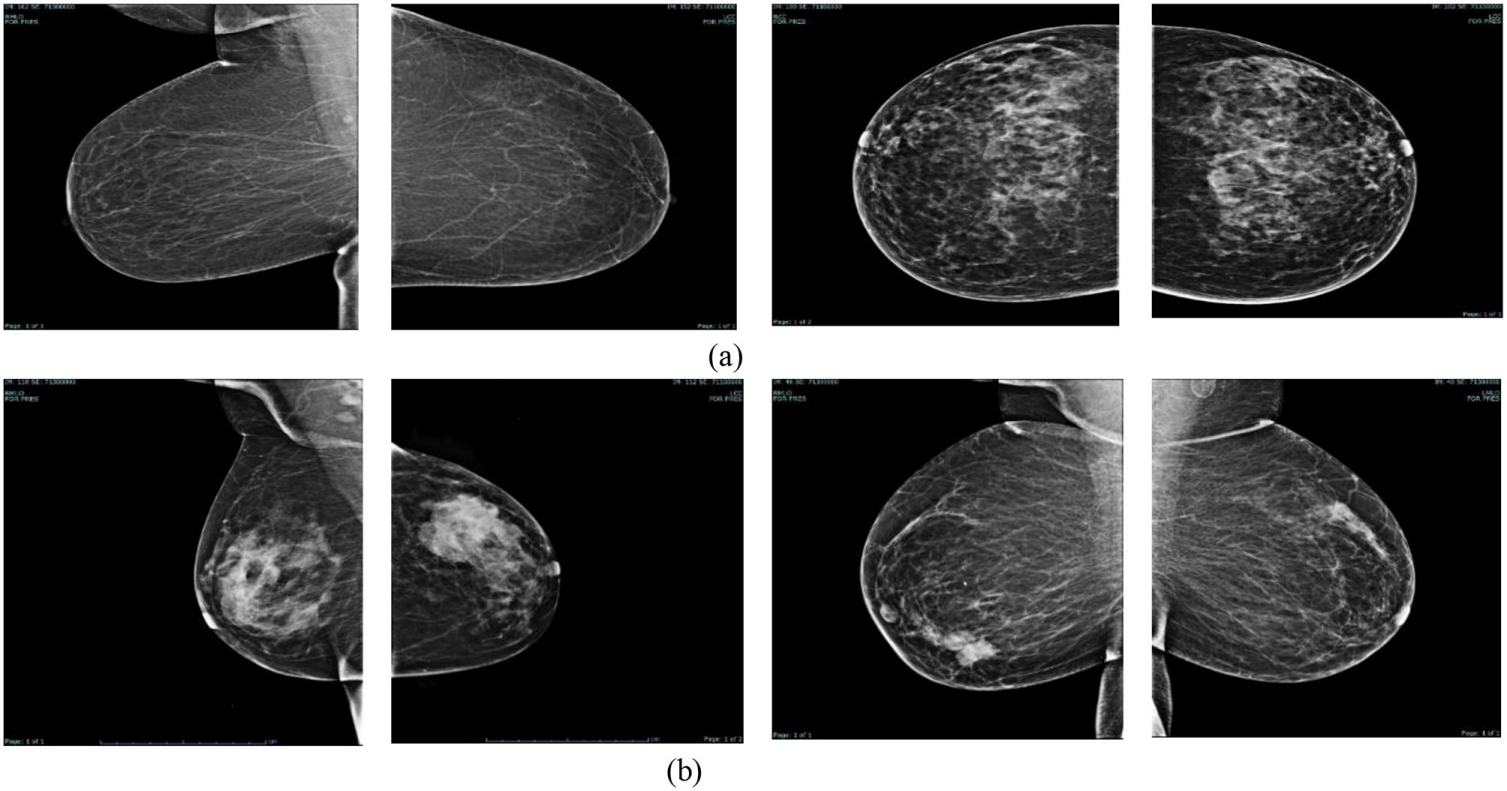
Samples of mammography breast images dataset. **(A)** Benign. **(B)** Malignant.

The mammography dataset used in this study was collected from King Abdullah University Hospital, Jordan University of Science and Technology, between early 2019 and late June 2024. The collection, segmentation, and diagnosis of images took 3 months, starting from July to September 2024, by clinicians from the hospital. It was named King Abdullah University Hospital Breast Cancer Mammography Dataset (KAUH-BCMD). The mammography dataset includes 7,205 images collected from 5,000 cases aged 18–75 years. The image size in JPG format is 720 × 720 pixels. Table 3 shows the distribution of images within each category. The images are divided into two groups: malignant and benign. Some of the images collected from the hospital are shown in Figure 2.

**Table 3:** Breast cases and the number of images in each case.

**Case**	**Number of images**
Benign	6,200
Malignant	1,005
Total	7,205

#### 3.1.2 Value of the dataset mammography

Screening mammography is the primary method for early identification of breast cancer since it is the only imaging modality that has consistently been shown to reduce mortality. Mammography can detect cancer up to 4 years before it develops clinically (Tabár et al., 2001). These systems, which use computer technology to identify abnormalities in mammograms (calcifications, masses, architectural distortions, etc.) that radiologists use as second opinion criteria, can be extremely useful in the early detection of breast cancer and contribute to a cost-effective reduction in the death rate among women with the disease (Sampat et al., 2005). This dataset of breast mammography pictures can be used to train machine learning algorithms capable of recognizing and detecting cancer.

#### 3.1.3 Materials and techniques used to collect data

Images were collected and archived by medical professionals from King Abdullah Hospital to verify and correct inaccurate diagnoses and to segment images into benign and malignant files. The device used for screening was a mammography machine from IMS Giotto, a subsidiary of GMM Group. The apparatus produces superior images despite a low signal-to-noise ratio. The dataset has 5,000 instances, each including 7,205 mammograms from two different perspectives (CC and MLO) of either the right or left breast. The preponderance of the benign images in our dataset can be attributed to screening programs provided to the general public by the hospital from which the cases were collected. The global standard for the transfer, preservation, and presentation of medical imaging data is known as Digital Imaging and Communications in Medicine (DICOM). Images were saved in DICOM format, a common format for mammography. Table 4 also provides a summary of the data connectors and how they work.

**Table 4:** Data assembly specifications.

**Specifications table**	
Subject area	Medicine
More specific subject area	Radiology and Imaging
Type of data	Images
Date of the scan	It is an identification code that distinguishes the records
Breast type	Left or right breast
Breast view	CC or MLO
Data format	JPG
Experimental factors	Every picture is categorized as either benign or malignant
Experimental features	Machine learning and deep learning models are developed using medical pictures to quickly and accurately classify and identify breast cancer
Data source location	King Abdullah University Hospital, University of Science and Technology, Irbid, Hashemite Kingdom of Jordan
Data accessibility	https://www.kaggle.com/mohammadaminalqasem

#### 3.1.4 Ethical considerations

Researchers acknowledge that patients have a right to privacy regarding their personal and medical affairs. To accomplish this, the researcher made sure that the hospital and patients were aware of the goals of the study. Furthermore, the disease status and personal information of each patient are confidential. Approval from the Institutional Review Board (IRB) was granted to Jordan University of Science and Technology (IRB number 21/171/2024). Furthermore, administrative approval from King Abdullah University Hospital (KAUH) was secured to examine the computerized medical records. This study utilized de-identified data for image interpretation and analysis, therefore negating the necessity for patient permission. This study adhered to the ethical criteria of the Helsinki Declaration, ensuring the protection of patient data privacy and confidentiality.

### 3.2 Data preprocessing

A crucial stage in the creation of any machine learning model, especially those that use images, is data preparation. The following steps were taken to make data preparation easier. For the dataset to be considered valuable, several requirements must be consistently met in recent years. The photos were compressed to 224 × 224 pixels using TensorFlow image processing techniques. This guarantees that every image sent to the model has consistent dimensions (Mavridis et al., 2024). Each image was assessed and deconvoluted by specialists at (KAUH). After dividing the image data by 255, pixel values were normalized to a range of 0–1. By keeping the input data consistent, this stage speeds up training (Huang et al., 2023). Because of their imbalance, the datasets were normalized using image preprocessing. We included fewer low-quality photos than high-quality ones to make sure the model treated both groups fairly and did not overestimate the importance of the former (Demircioglu, 2024). To assess the models, the dataset is finally split into three sections: 10% for testing, 10% for validation, and 80% for training. This enables us to assess the model's performance using new data.

This section provides an overview of the preprocessing techniques used to make the dataset more suitable before fitting into the Residual Depth-wise Network (RDN) model.

#### 3.2.1 Adaptive CLAHE (contrast limited adaptive histogram equalization)

Adaptive CLAHE is a technique that improves visual contrast by altering it locally rather than globally. This method solves the limitations of standard Histogram Equalization (HE), which can result in over-enhancement in some regions and loss of information in others.

##### 3.2.1.1 Adaptive histogram equalization

In image processing, histogram equalization (HE) is a technique used to improve contrast. The fundamental idea behind histogram equalization (HE) is to modify an image's pixel values in order to provide a more consistent distribution across all potential values. The first stage in histogram equalization (HE) is to create the histogram of the image, which visually represents the frequency of each pixel value in the image (Rao, 2020). The pixel values are then reallocated to provide a more uniform distribution of the histogram across the entire spectrum of possible values (Doshvarpassand et al., 2022). The histogram equalization approach consists of two components:

1. Calculating the cumulative distribution function (CDF) of the image.
2. Using the CDF to transform the pixel values of the image into a new range (Majeed and Isa, 2021). The histogram data are aggregated from the leftmost bin to the rightmost bin to derive the cumulative distribution function (CDF).
3. The CDF value is multiplied by the maximum pixel value, and the outcomes are rounded to the nearest integer, therefore transforming the pixel values to the new range.

HE can be used for both color and grayscale images, which makes it suitable for mammogram-type breast cancer images. The following formula applies to HE. Let *H*(*k*) be the frequency histogram of the input image x, which has *n* rows and *m* columns. Let k be in the range from 0 to I-1, where I is the number of intensity levels. Equation 1 used to calculate the normalized frequency histogram.


(1) $$\begin{array}{l} N\left(k\right)=\frac{H\left(k\right)}{n\times m} \end{array}$$

Utilize Equation 2 the normalized histogram's cumulative distribution function (or CDF) is calculated.


(2) $$\begin{array}{l} CDF\left(j\right)=\overset{j}{\underset{k=0}{\sum }}N\left(k\right),\;for\;j=0,\;1,\;\ldots ,\;I-1 \end{array}$$

Employ Equation 3 to determine each pixel's new intensity values.


(3) $$\begin{array}{l} P=round\left(I-1\right)\times CDF\left(x\right) \end{array}$$

Assign a new intensity value to each pixel in the specified image. The HE formula converts the original image intensity levels into new values utilizing the cumulative distribution function of the histogram. Distributing the intensity values throughout the full spectrum enhances the image's contrast (Chen et al., 2023).

##### 3.2.1.2 Adaptive CLAHE

CLAHE limits contrast enhancement by initially truncating the histogram at a specified clip limit and subsequently redistributing the clipped values among the bins. The approach is adaptable since it handles each local region (or window) of the image separately. CLAHE can improve local contrast by breaking the image into smaller tiles and applying histogram equalization on each one (Oza et al., 2024). The steps for CLAHE can be described as follows:

- Step 1: Create tiny overlapping areas (tiles) in the image.
- Step 2: Compute the histogram for each region.
- Step 3: Clip the histogram to a predefined limit and redistribute the clipped pixel values evenly.
- Step 4: Compute the Cumulative Distribution Function (CDF) and apply the equalization to map the pixel values.
- Step 5: Interpolate between adjacent tiles to avoid artifacts.

The CLAHE transfer function is derived from the cumulative histogram, where the clipping value *C* is applied to limit the amount of contrast stretching. The transformation function for CLAHE in a local region L is defined as:


(4) $$\begin{array}{l} f\left(g\right)=\frac{L-1}{n}\;\overset{L}{\underset{g=0}{\sum }}H\left(g\right) \end{array}$$

where *n* is the total number of pixels in the window, and *H*(*g*) represents the region's pixel value histogram. CLAHE outperforms classic AHE by regulating the level of enhancement via the clip limit *C* and eliminating artifacts such as over-amplified noise (Roy et al., 2024).

#### 3.2.2 Inverted

Inverted Image Processing refers to the process of reversing an image's color values. Each pixel's intensity is deducted from the highest intensity value (255 in an 8-bit picture). Inverting an image is beneficial for some image analysis tasks, such as highlighting darker spots in light backdrops or reversing the effects of underexposure in photos. The equation for image inversion is:


(5) $$\begin{array}{l} I_{inverted}\left(x,y\right)=255-I\left(x,y\right) \end{array}$$

where *I*(*x, y*) represents the original pixel value at coordinates (*x, y*), and *I*_*inverted*_(*x, y*) is the inverted pixel value (Yang, 2008).

#### 3.2.3 High-boost filtering

High-boost filtering is a technique for sharpening photographs that amplifies high-frequency components while retaining low-frequency information. It is an improvement on high-pass filtering in that instead of deleting all low-frequency components, we keep them and amplify the high frequencies for a sharper output.

##### 3.2.3.1 High-pass filters

A high-pass filter removes low-frequency components (such as smooth or steady areas) while retaining high-frequency components (edges and fast intensity variations) from an image. The fundamental equation for a high-pass filter is:


(6) $$\begin{array}{l} High\;Pass\left(f\left(x,y\right)\right)=f\left(x,y\right)-Low\;Pass\left(f\left(x,y\right)\right) \end{array}$$

where *f*(*x, y*) is the original image and *Low Pass*(*f*(*x, y*)) is the low-pass filtered image (smoothing operation like Gaussian blur) (Esmaeilzehi et al., 2024).

##### 3.2.3.2 High-boost filtering

High-boost filtering enhances the high-frequency components while keeping some of the low-frequency information. This is done by applying a high-pass filter with a gain factor *A*. The high-boost filter is given by:


(7) $$\begin{array}{l} High\;Boost=A\cdot f\left(x,y\right)-Low\;Pass\left(f\left(x,y\right)\right) \end{array}$$

To balance the filter, we can rewrite it as:


(8) $$\begin{array}{l} High\;Boost=\left(A-1\right)\cdot f\left(x,y\right)+High\;Pass \end{array}$$

Where *A* is the amplification factor. By adjusting *A*, the intensity of sharpening can be controlled. This formula illustrates the operation of high-boost filtering: the original image's high-frequency components are amplified by multiplying it by A, and the edges are further enhanced by deleting the low-pass image (Soniminde and Biradar, 2024).

**Figure 3:**
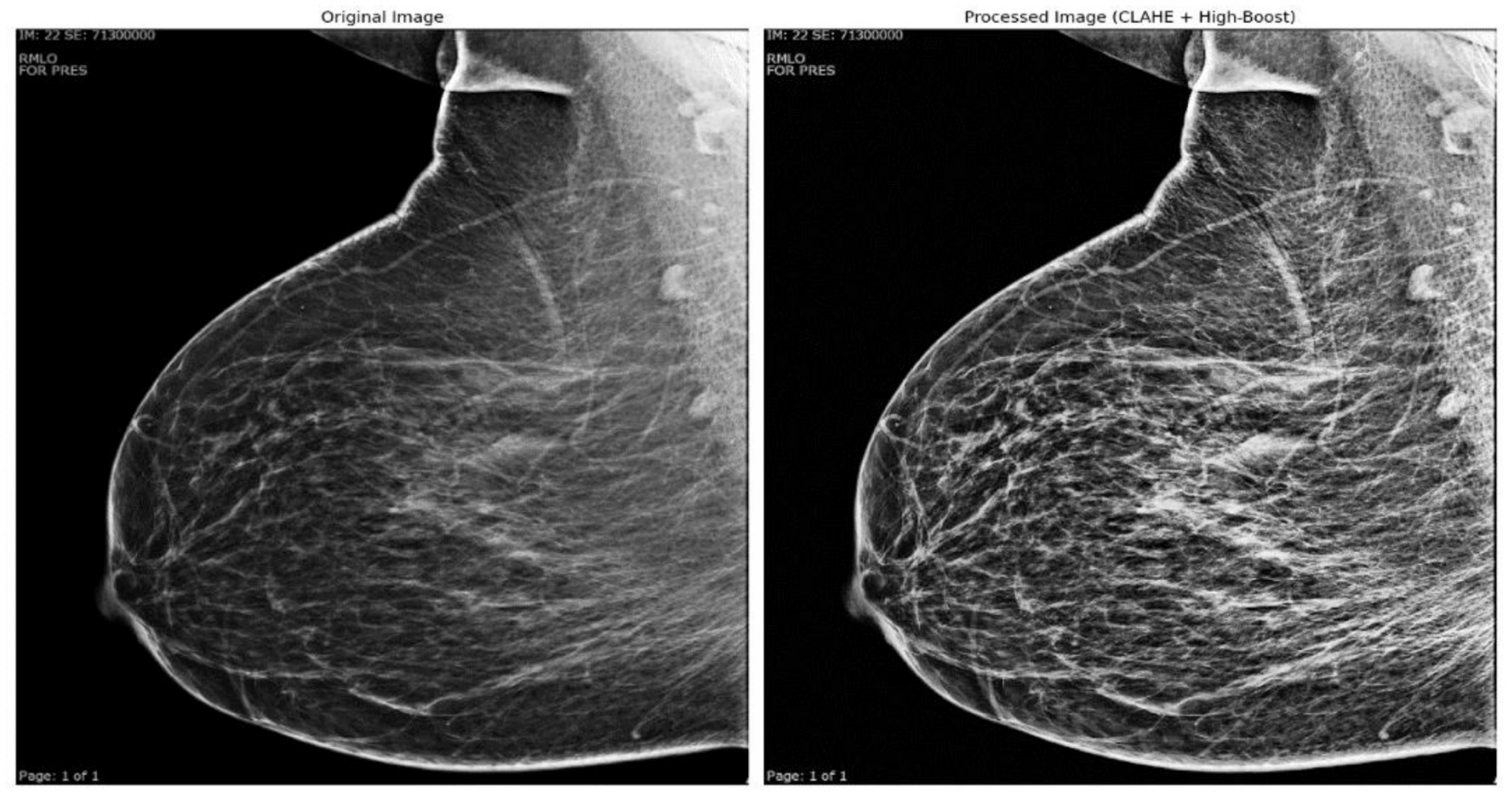
Original grayscale medical image pre-processed using CLAHE technology.

Figure 3 shows the original grayscale medical image that was preprocessed using CLAHE (Contrast-Limited Adaptive Histogram Equalization). The stronger contrast in the original image may have made it difficult to see fine details within the tissue. In comparison, the CLAHE-processed image has higher local contrast, making it easier to detect details and textures. CLAHE improves contrast in specific areas of the image. It also improves the overall balance of the image by highlighting smaller details without exaggerating background noise.

**Figure 4:**
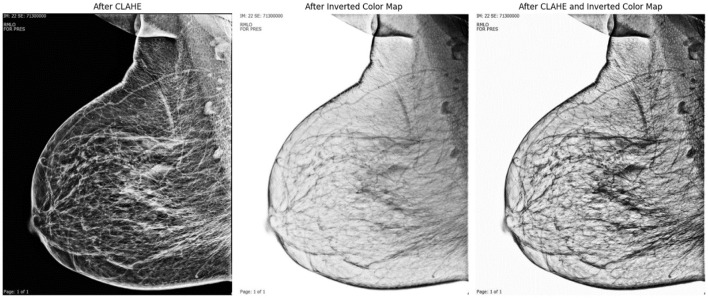
Image processing stages.

Figure 4 shows the post-processing stages. The After CLAHE image continues to emphasize detail by increasing contrast. The After Inverted Color Map image reflects pixel intensity, making previously dark areas appear lighter and vice versa. This reflection can sometimes help you see certain features or abnormalities more clearly. CLAHE and reflection are combined in the After CLAHE and Inverted Color Map picture. This image shows details highlighted using different highlighting techniques, which aids in visual inspection. These images illustrate how different methods of medical image processing can help clarify distinct components for greater understanding.

**Figure 5:**
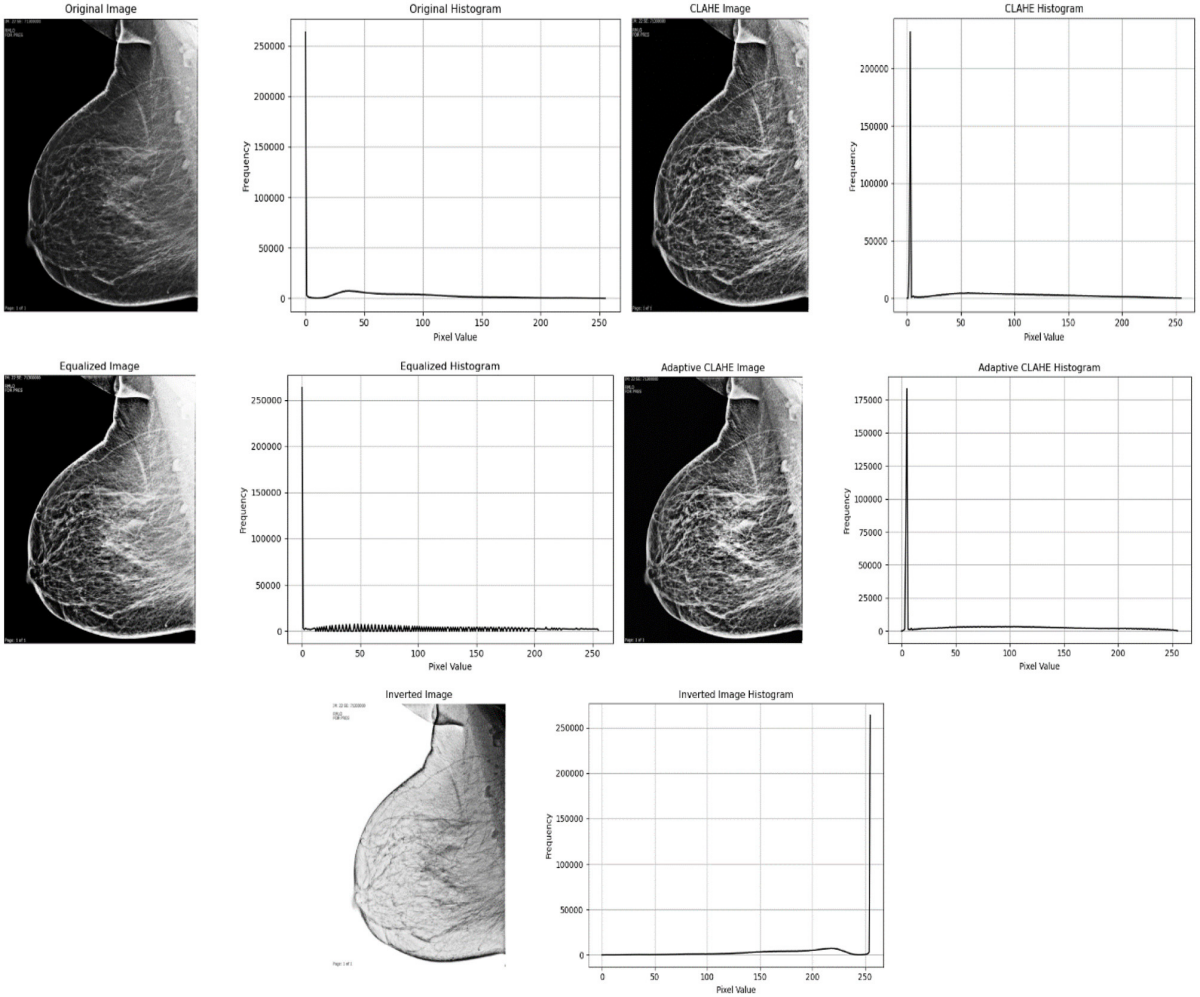
Below is a grayscale medical image with enhanced resampling using different methodologies and accompanying visual representations.

Figure 5 demonstrate a grayscale medical image (mammogram) with improved rephrasing using various methodologies and accompanying visual representations. The original image and histogram indicate that there are more dark hues than light ones. Histogram Equalization improves the overall contrast of an image by spreading out the brightness levels more evenly, resulting in a balanced histogram. CLAHE (Contrast Limited Adaptive Histogram Equalization) increases an image's local brightness and contrast while making noise less visible. The histogram reveals a somewhat different distribution of brightness levels. A separate Adaptive CLAHE is utilized to emphasize certain locations, with its histogram altered to fit. Finally, Image Inversion adjusts the brightness of the pixels, making dark areas appear lighter.

### 3.3 Pre-trained models

Pre-trained deep learning architectures have been developed and improved upon using large datasets. Consequently, they can extract extensive and meaningful data from the input. These models provide a strong basis for many applications, such as picture classification, language translation, and object recognition. Pre-trained models can significantly reduce training time and effort when working with tiny or new datasets because their performance can be adjusted to the desired outcome by “training by optimization” or “fine-tuning.” This article classifies breast cancer mammography images using seven pre-trained models and contrasts them with the methodology described in the study.

VGG16 might be a deep convolutional neural structure consisting of 16 weight layers, including 3 fully associated layers and 13 convolutional layers. After ReLU enactments and max-pooling layers to reduce spatial measurements, it creates a 224 × 224 × 3 (RGB) input image using various convolutional components. With 1,000 neurons and a softmax actuation, the ultimate yield layer is suitable for categorizing images into 1,000 groups, as demonstrated by the ImageNet dataset (Belaid and Loudini, 2020).

VGG19 the 19 layers of the VGG19 model consist of 16 convolutional layers, 3 pooling layers, and output layers. The model's simple, successive layer structure preserves consistency and symmetry while enabling it to identify complex patterns and features in images. It uses tiny (3 × 3) filters to capture minute details in photos (Simonyan and Zisserman, 2014).

MobileNetV2 has 53 layers, consisting of rearranged remaining squares with depthwise divisible convolutions and direct bottlenecks. It takes an input picture of 224 × 224 × 3 (RGB) and produces a yield reasonable for the assignment, such as a 1,000-class softmax for ImageNet classification (Xiaolong et al., 2020). Its plan centers on proficiency, making it perfect for versatile and embedded devices.

ResNet50 consists of 50 layers, with 1 × 1, 3 × 3, and 1 × 1 convolutions for the remaining bottleneck pieces. A 224 × 224 × 3 (RGB) input image is used to generate a 1,000-class softmax forecast for ImageNet classification, or it can be modified for other tasks (Hossain et al., 2022).

InceptionV3 could be a 48-layer deep learning CNN designed to effectively capture highlights at various scales using Beginning modules. Concatenation follows the parallel application of 1 × 1, 3 × 3, and 5 × 5 convolutions with max pooling in each module. input 299 × 299 × 3 (RGB) image. For ImageNet classification, produce a 1,000-class softmax; it can also be used for other tasks. It is renowned for modifying computational productivity, profundity, and width (Sharma et al., 2023).

Xception (Extreme Inception) is a CNN with 36 convolutional layers, organized into passage, center, and exit stream modules. It replaces standard convolutions with depthwise distinct convolutions to progress effectiveness and execution. Input picture of 299 × 299 × 3 (RGB). Yield 1000-class softmax for ImageNet classification or versatile for other assignments. Xception offers a more profound, more effective engineering motivated by Initiation, optimized for tall performance (Salim et al., 2023).

DenseNet121 121 layers arranged in thick squares, maybe a CNN, where each layer is connected to another layer within the same piece. Compared to traditional systems, this structure reduces the amount of parameters and emphasizes reuse. ^**^Input: 224 × 224 × 3 (RGB) image. ^**^Yield: flexible for other tasks or 1,000-class softmax for ImageNet classification. DenseNet121 is renowned for its effectiveness; it uses fewer parameters while maintaining high accuracy across the thick network (Kateb et al., 2023).

### 3.4 Proposed residual depth-wise network (RDN) models

**Figure 6:**
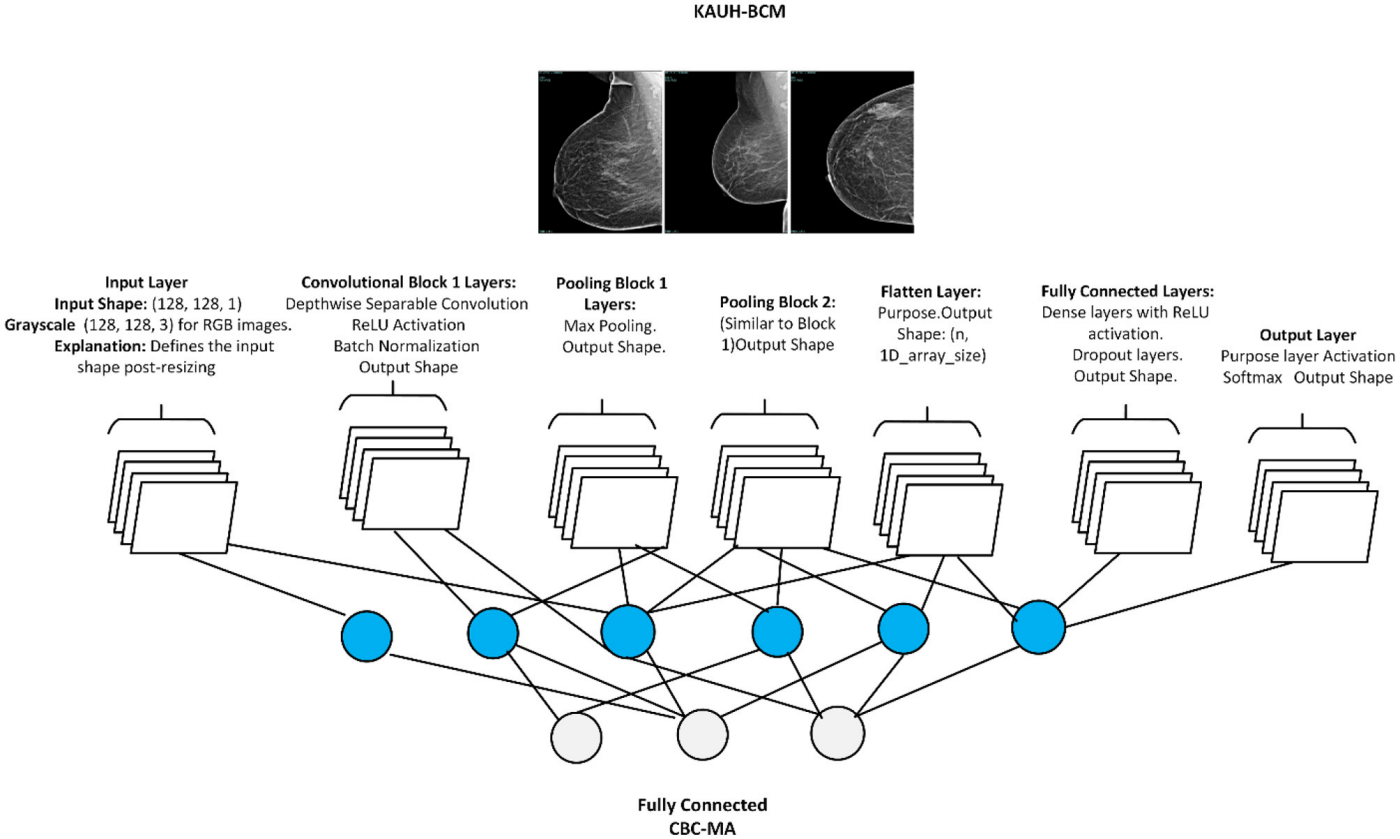
Residual depth-wise network (RDN).

The proposed method for image enhancement and analysis images involves a systematic approach that improves the quality of the images, expands the collection, and emphasizes key features. This process gets images ready for better analysis, making sure the data given to the model is clear and uniform. After preparing the data, we use histogram analysis and other methods like Contrast Limited Adaptive Histogram Equalization (CLAHE) and Sobel edge detection to improve and separate important areas, making it easier to identify features. Figure 6 presents the Residual Depth-wise Network (RDN) Model architecture.

We recommend employing a Convolutional Neural Network (CNN) architecture specifically tailored to handle the processed images for the modeling phase. This layer configuration demonstrates the operation of this architecture. Each stage in the model, particularly the convolution and pooling layers, benefits from the rich and diverse feature sets produced during preprocessing, enhancing robustness and accuracy in subsequent tasks.

#### 3.4.1 Implementation note-residual depth-wise network (RDN) model layers

- Input Layer to serve as the passage point for pictures into the organize. Input: An RGB picture of measuring 128 × 128 pixels. Yield Shape: (128, 128, 3) representing 128 × 128 spatial dimensions with 3 color channels (R, G, B).
- Convolutional Layers to detect feature patterns by sliding filters (kernels) across the image. Typically employs filters of size 3 × 3, with the number of filters increasing in deeper layers to capture complex features. Layer: Conv2D (32, 3, 3, activation = “relu”). Output Shape: Input: (128, 128, 3). Output: (126, 126, 32), after applying the 32 filters and reducing spatial dimensions by 2 pixels on each side.
- ReLU (Rectified Linear Unit) Activation, non-linearity to aid the network in learning complex patterns. Outputs: zero for negative values and retains positive values unchanged, mitigating issues with gradient vanishing.
- Layer DepthwiseConv2D with specific kernel size (3, 3). Input Shape: (128, 128, 3) for an RGB image. Output Shape: After applying depthwise filters, the spatial dimensions change based on kernel size and padding, but the number of channels remains the same per individual channel convolution. For example, with padding set to “same,” the output shape would remain (128, 128, 3), but it could change based on padding and stride configuration.
- Batch Normalization of the output from the previous layer, expediting training and providing some regularization. Output Shape: Remains unchanged from input.
- Pooling Layers (Max Pooling): to reduce spatial dimensions, enhancing computational efficiency while focusing on prominent features, utilizes a 2 × 2 filter to halve each dimension. Layer: MaxPooling2D (pool_size = (2, 2)). Output for input: (126, 126, 32) is (63, 63, 32). Achieving down sampling of the spatial dimensions.
- Dropout Layer: Mitigates overfitting by randomly setting a fraction of the input units to zero during training. Commonly set between 0.2 and 0.5 for the dropout rate. Layer: Dropout (0.5). Output Shape: Unchanged from input.
- Flatten Layer: Converts 2D feature maps into a 1D vector for input into the dense layers. yielding a 1D vector containing all features.
- Fully Connected (Dense) Layer: Executes high-level reasoning based on features extracted by convolutional and pooling layers. The first dense layer typically has more units to capture complex interactions, while the last layer's unit count aligns with the number of output classes.
- Output Layer (sigmoid for Classification): Produces probabilities for each class in a classification task. Layer: Dense (num_classes, activation= “softmax”) for classification. where each value denotes the probability of each class.

Given a 128 × 128 RGB input image and employing standard layer configurations, the transformations of shapes would progress as follows in Table 5.

**Table 5:** Layer configurations.

**Layer**	**Output shape**
Input layer	(128, 128, 3)
Conv2D (32 filters, 3 × 3)	(128, 128, 32)
DepthwiseConv2D (3 × 3)	(128, 128, 32)
Conv2D (32 filters, 1 × 1)	(128, 128, 32)
Shortcut (Conv2D, 1 × 1)	(128, 128, 32)
Add Layer (Skip connection)	(128, 128, 32)
Conv2D (64 filters, 1 × 1)	(128, 128, 64)
DepthwiseConv2D (3 × 3)	(128, 128, 64)
Conv2D (64 filters, 1 × 1)	(128, 128, 64)
Flatten	(128 × 128 × 64)
Dense (1 unit, Sigmoid)	(1)

Each layer builds upon the previous one, progressing from basic feature extraction to high-level pattern recognition and classification.

### 3.5 Evaluation measures

Accuracy was calculated by dividing the fraction of accurately detected data instances by the total number of occurrences. This allowed us to measure the model's efficacy. The accuracy equation is presented below. True Positive (TP) refers to data that has a positive impact and is appropriately classed with the target label. True Negative (TN) refers to data with a negative impact that is appropriately classified as such by the target label. When False Positive (FP) data are incorrectly labeled as positive, the target label suffers. False Negative (FN) statistics, on the other hand, are properly classified as negative and carry the required classification. The evaluation measures are supplied in the equations from Equations 9–13.

- Accuracy: calculate the percentage of accurately identified data by combining true negatives *TN* and true positives *TP*.

(9) $$\begin{array}{l} Accuracy=\frac{TP+TN}{TP+TN+FP+FN} \end{array}$$

- Precision: calculate the true positive ratio by adding the true positives and false positives *FP* together.

(10) $$\begin{array}{l} Precision=\frac{T_{p}}{T_{p}+F_{p}} \end{array}$$

- Recall (Sensitivity): evaluate the proportion of genuine positives to the total of true positives plus false negatives *FN*.

(11) $$\begin{array}{l} Recall=\frac{T_{p}}{T_{P}+F_{N}} \end{array}$$

- Specificity: is a metric used to assess how well categorization models perform, particularly when working with binary data or in the medical industry.

(12) $$\begin{array}{l} Specificity=\frac{TN}{TN+FP} \end{array}$$

- F1 Score: Measure harmonic means of precision and recall.

(13) $$\begin{array}{l} F_{1}-Score=2\times \frac{precision\times Recall}{Precision+Rrecall} \end{array}$$

The number of cases correctly categorized as positive is referred to as “true positives” (TP). True negatives (TN) are accurate estimations of negative cases. Positive cases that were wrongly found are known as false positives or FPs. False negatives, or FN, refer to the number of cases that were incorrectly projected to be in the negative group. The percentage of correct forecasts including true positives and true negatives to all predictions is known as accuracy (Yacouby and Axman, 2020).

### 3.6 Computing environment

The coding and development operations for this work were carried out on a computer with the following specifications:

- Processor: Intel Core i5-12600
- RAM: 32 GB
- Operating System: [Windows 11]
- Other Specifications: [GPU RTX 4070]

### 3.7 Limitations

Numerous constraints must be acknowledged when gathering data from a hospital. To safeguard patient privacy and adhere to regulatory regulations, it is imperative to omit identifying information, clearly articulate the study's aim, and secure the data against unauthorized access. Ethics committees and pertinent authorities must also grant the requisite authorization. Moreover, data minimization principles must be observed, necessitating the retention of only critical information and the specification of its retention duration before destruction. AI models are vulnerable to biases stemming from skewed or unrepresentative datasets. This study utilized a substantial and varied dataset of mammography pictures from patients aged 18–75 with differing breast densities; yet, inherent biases may persist. The underrepresentation of specific demographic groups may affect the model's generalizability. A significant problem of AI-based systems in healthcare is their “black box” characteristic, which constrains trust and interpretability. The incorporation of explainable AI (XAI) methodologies is essential to mitigate this constraint.

## 4 Results and discussion

A method using a multi-fusion processing approach is proposed. Multi-fusion image processing technique is used to improve feature extraction, including inverse, CLAHE adaptive histogram equalization, and high-boost filtering. The classification model uses a proposed residual depth network (RDN), which integrates residual learning with depth-separable convolutions to enhance computational efficiency and accuracy. The process also involves training seven pre-trained neural network models: MobileNetV2, VGG16, VGG19, ResNet50, InceptionV3, Xception, and DenseNet121 while evaluating their performance against the proposed RDN model using six metrics. Precision, Recall, Sensitivity, and Specificity F1-score Precision Validation.

Table 6 shows the performance characteristics of deep learning models that classify breast cancer mammogram images into benign and malignant categories. Accuracy, precision, recall (sensitivity), specificity, F1 score, and cross-validation accuracy are the metrics used to evaluate the models' performance. The RDN model outperformed the other trained models when classifying breast cancer mammography pictures. The model demonstrated its efficacy in effectively categorizing benign and malignant patients with a 97.82% accuracy rate, 96.55% precision, 99.19% recall, and 96.45% specificity. With a 97.85% F1 score, accuracy and recall are strongly balanced, indicating that CBC-MA is reliable. DenseNet121 and VGG16 are excellent choices for classifying breast cancer images. DenseNet121 achieved a high accuracy of 95.24% and an exceptional recall of 99.84%. Their results showed that VGG16 performed excellently, achieving an accuracy of 95.48% and a perfect recall of 100%, meaning it correctly identified all malignant cases. Therefore, these two models are recommended as a secondary choice after the Residual Depth-wise Network (RDN).

**Table 6:** Performance comparison of deep learning models for breast cancer classification using KAUH-BCMD.

**Model**	**Accuracy**	**Precision**	**Recall (sensitivity)**	**Specificity**	**F1 score**	**Validation accuracy**
MobileNetV2	92.90	87.89	99.52	86.37	93.39	93.47
VGG16	95.48	91.58	1.00	90.81	95.51	95.32
VGG19	77.26	80.99	71.29	93.23	75.86	75.48
ResNet50	87.82	92.69	82.02	93.55	87.08	85.16
InceptionV3	62.58	67.69	48.23	76.94	56.29	64.27
Xception	93.31	89.29	98.39	88.23	93.61	93.71
DenseNet121	95.24	91.43	99.84	90.65	95.41	95.24
RDN	97.82	96.55	99.19	96.45	97.85	96.20

MobileNetV2, Xception, and ResNet50 have shown good performance. MobileNetV2 achieved commendable performance with 92.90% accuracy and 99.52% recall, demonstrating its strong ability to identify fraudulent cases. The F1 score of 93.39% indicates a strong balance between accuracy and recall. Xception showed excellent performance with 93.31% accuracy 98.39% recall, and 88.23% specificity, reflecting a well-balanced effectiveness. ResNet50 achieved 87.82% accuracy, 92.69% precision, and 93.55% specificity, demonstrating its effectiveness in reducing false positives.

On the other hand, among all the models, VGG19 and InceptionV3 performed the worst. VGG19 achieved an accuracy rate of 77.26%. While the specificity of 93.23% was quite high, the recall rate of 71.29% indicates difficulties in correctly identifying malignant individuals. When it comes to accuracy 62.58% and recall 48.23%, InceptionV3 performed the worst, making it unreliable for identifying cancer patients. The results show that the proposed RDN model outperformed other models in classifying breast cancer mammogram images on several metrics, such as accuracy, precision, recall, and F1 score. It proved the best choice due to its exceptional ability to distinguish between cancerous and benign cases.

**Figure 7:**
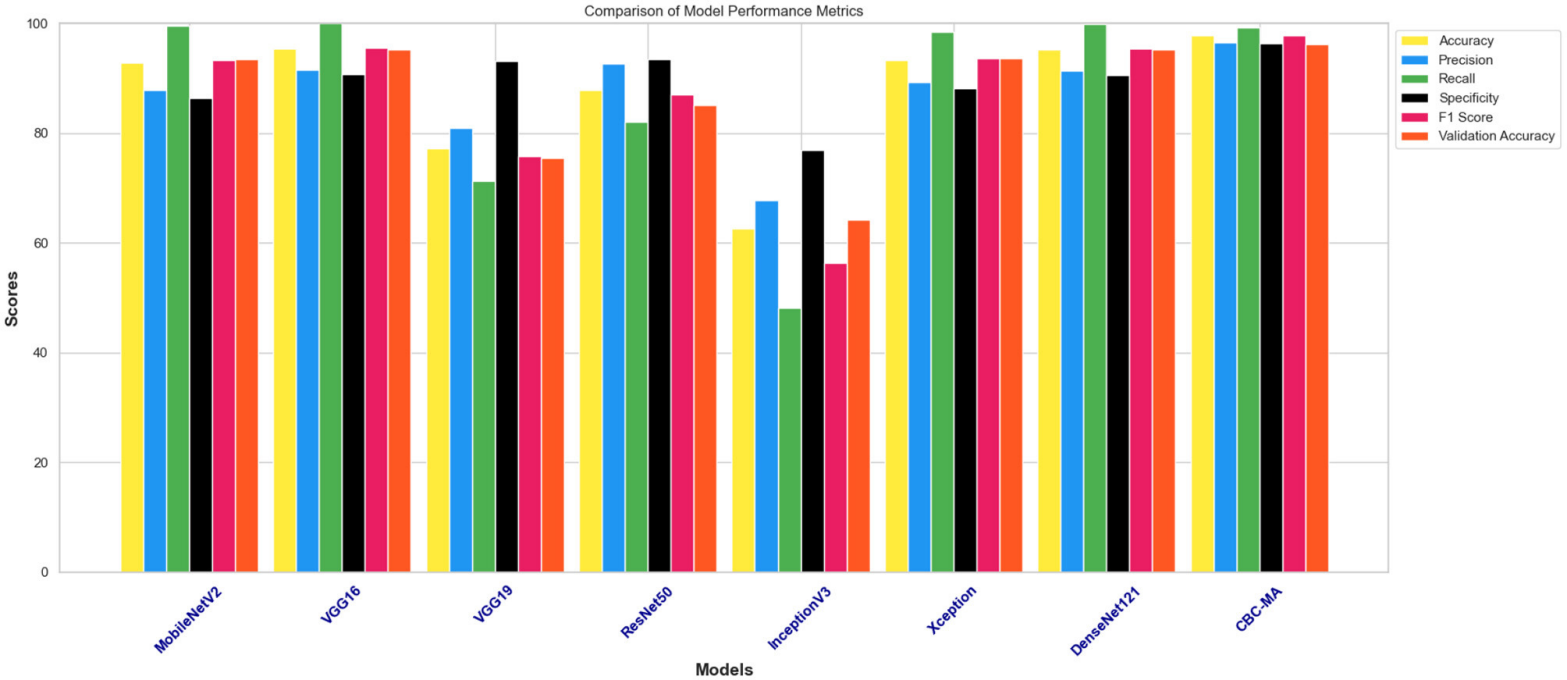
Comparing performance metrics of different models.

Figure 7 illustrates the performance graphs of eight distinct deep learning models MobileNetV2, VGG16, VGG19, ResNet50, InceptionV3, Xception, DenseNet121, and CBC-MA employed for breast cancer classification using mammography pictures. The models are assessed using six performance metrics: accuracy, precision, recall, specificity, F1 score, and validation accuracy. Each color in the graph represents a unique metric, facilitating a more comprehensive comparison of the models.

The RDN model performs excellently in classifying breast cancer images, with the best accuracy, specificity, recall, F1 score, and validation accuracy, making it the most effective model. The InceptionV3 model has the poorest performance, especially in recall and validation accuracy, which may lead to the under-identification of some malignant cases. Models such as VGG16 and DenseNet121 show excellent performance and balance, with VGG16 achieving 100% recall and demonstrating its ability to detect all malignant cases without missing any. Models that achieve balance across all metrics are preferred for clinical use because they generate accurate and consistent results; therefore, RDN appears to be the best choice.

**Figure 8:**
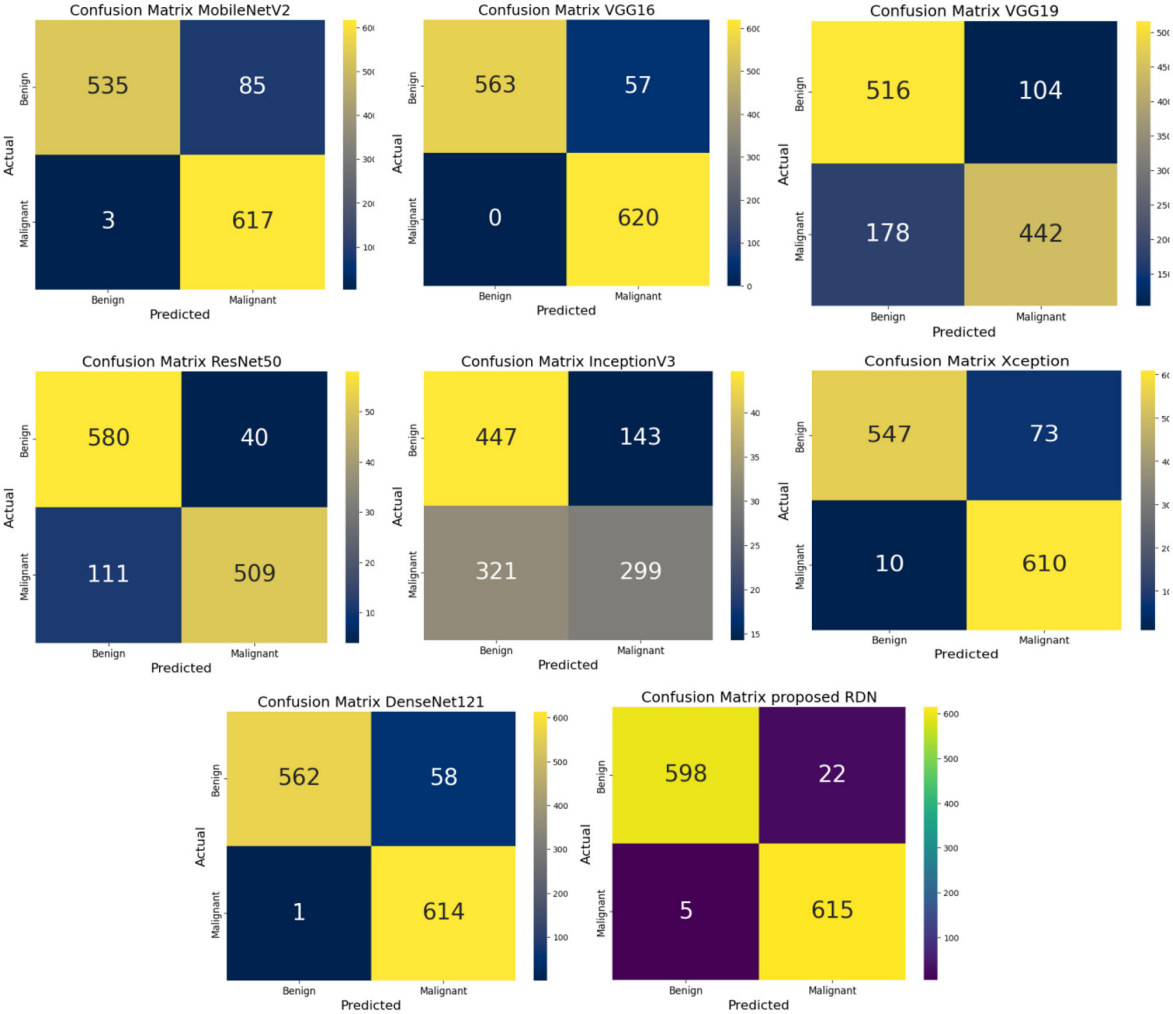
Comparing confusion matrices for different models.

According to the confusion matrices shown in Figure 8, the RDN model performed better than any other model, achieving a great balance between recall and precision with few errors in classifying malicious and benign data. Both DenseNet121 and VGG16, which replaced RDN, showed a great ability to classify samples accurately, albeit with small mistakes. On the other hand, InceptionV3 performed the worst, making many errors while trying to distinguish between malicious and benign data, making it generally unsuitable for classification in this situation. To improve classification accuracy, these matrices can identify areas for improvement and evaluate the model's performance.

**Figure 9:**
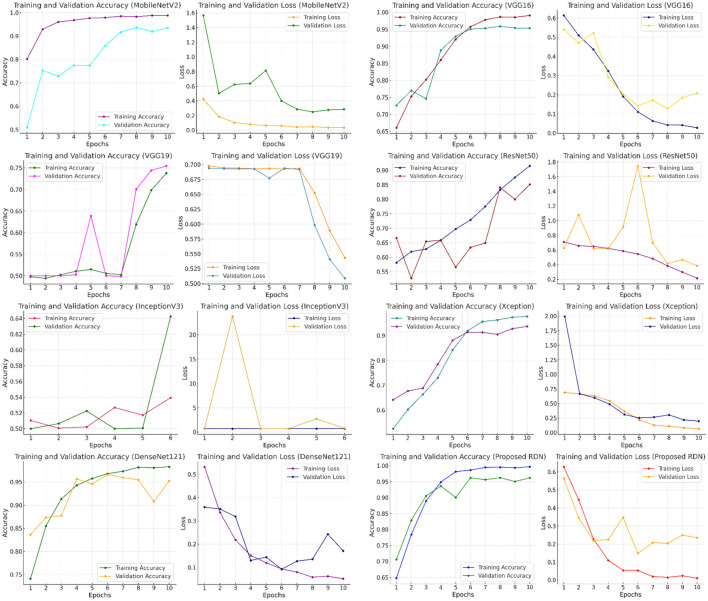
Comparing the performance of different deep learning models in terms of accuracy, training loss, and evaluation.

To illustrate the performance of different models, Figure 9 displays graphs that track training and evaluation accuracy and loss throughout a large number of training epochs. By examining these graphs, one can determine overfitting or inadequate generalization, as well as how well the model learns from the data and gets better. By comparing the performance of models like MobileNetV2, VGG16, VGG19, ResNet50, InceptionV3, Xception, DenseNet121, and the suggested RDN, the graphs help users make better decisions about which model to use by identifying which one offers the best balance between learning loss and training and evaluation accuracy. The residual dense network, or RDN, performed admirably. The high training accuracy (99.7%) in the graphs indicates that the model successfully absorbed the training data. Additionally, the excellent evaluation accuracy of the model (96.2%) showed how well it translated to new data.

### 4.1 Testing the model in clinical settings

Physicians have provided positive feedback on the use of the model for evaluating mammograms. After applying this model in 20 cases, the clinicians found it to be a great tool in emphasizing clinical decisions, and they have observed promising results. Of the 20 cases, 15 were correctly diagnosed, with the other 5 cases showing variabilities that needed further evaluation and investigation.

The model's ability to quickly scan mammographic images and to determine areas of concern provided clinicians with a second supporting opinion and increased their confidence in reaching the correct diagnoses. This feedback emphasized the system's efficiency, as it greatly decreased the time spent on manual image reading, allowing for fast decisions on each case, reducing clinician workload, and improving patient care and outcome. Also, clinicians noted that the model is highly accurate in detecting subtle changes that may have been under-evaluated by them. This accuracy was especially important in borderline findings, where additional investigations were needed to confirm or exclude certain diagnoses. The integration of the model into the clinical investigation of the cases can further enhance its utility and cover the deficiencies encountered in diagnosis by either the model alone or by the clinician alone. Overall, that feedback reflected strong recommendations of using such a model in enhancing diagnostic accuracy and improving clinical practice; due to its potential to improve patient care, clinicians thought that its use would jump rapidly with the trust in its performance.

## 5 Conclusion

The main contribution of this study is to present an open-source dataset for breast cancer diagnosis using mammogram images collected by specialized physicians from King Abdullah University Hospital in the Hashemite Kingdom of Jordan, referred to in this paper as (KAUH-BCMD). This dataset is the first to be collected in the Hashemite Kingdom of Jordan, providing AI researchers with a reliable dataset to increase diagnostic efficiency. Moreover, multiple fusion methods, such as high-resolution enhanced filtering and contrast-limited adaptive histogram equalization (CLAHE), have been proposed to improve image quality. We have constructed a unique residual depth network (RDN) to enhance the accuracy of breast cancer detection. The proposed RDN model has been compared with several prominent models, including MobileNetV2, VGG16, VGG19, ResNet50, InceptionV3, Xception, and DenseNet121. The RDN model showed superior performance, achieving 97.82% accuracy, 96.55% precision, 99.19% recall, 96.45% specificity, 97.85% F1 score, and 96.20% validation accuracy. This indicates its effectiveness in diagnosing breast cancer patients.

In the future, we aspire to integrate the pertinent mammography dataset with breast ultrasound data to develop a multimodal CAD system for breast cancer. The next version featuring additional photos will enhance balance and clarity, hence assisting researchers in breast cancer detection systems. Furthermore, according to prior and current studies, computer vision models can benefit patients and healthcare practitioners. Consequently, it will enhance temporal efficiency, augment diagnostic efficacy, and expedite breast cancer identification.

## 6 Future works

In the future, we hope to combine relevant mammography datasets with breast ultrasound data to create a multimodal CAD system for breast cancer. The next iteration, with more photos, will improve the balance and clarity, assisting researchers in breast cancer detection systems. Another intriguing approach is to use federated learning frameworks, which enable decentralized data from several sources (for example, mammography and ultrasound centers) to build a single model without sharing raw data. However, in future work, we intend to include more databases, such as MIAS, INbreast, DDSM, and CBIS-DDSM, to increase the model's generalizability and assess it across datasets from various populations. This will help the model function better in practical circumstances. Furthermore, recent and present research suggests that computer vision models can help both patients and healthcare practitioners. As a result, they will save time, improve diagnostic accuracy, and speed up breast cancer detection.

We plan to collaborate with radiologists and healthcare centers to test the model using real data taken from daily clinical practices. We will collect expert feedback on usability and accuracy to improve the model and ensure its alignment with healthcare sector needs. To improve performance, the proposed model (RDN) will be tested using advanced architectures such as vision transformers (ViT) and attention mechanisms.

## Data Availability

The datasets presented in this study can be found in online repositories. The names of the repository/repositories and accession number(s) can be found at: https://www.kaggle.com/mohammadaminalqasem.
